# Assessment of an automated telesurveillance system on the incidence of serious falls in nursing homes (TELEHPAD): Randomized controlled trial

**DOI:** 10.1016/j.jarlif.2025.100025

**Published:** 2025-09-09

**Authors:** Abdoul Razak SAWADOGO, Jean-François NYS, Caroline GAYOT, Gilles KEHOUA, Achille TCHALLA

**Affiliations:** a Université de Limoges; Laboratoire Vie Santé UR 24134 | Vieillissement, Fragilité, Prévention, e-Santé | Institut OMEGA HEALTH chez Université de Limoges & CHU de Limoges, 33 rue François Mitterrand BP 23204 - 87032 Limoges, France; b Unité de Recherche Clinique et d’Innovation (URCI) en Gérontologie, Pôle Gérontologie Clinique, 2 Avenue Martin, 87042 Limoges, France; c Gérontopôle Nouvelle Aquitaine, **VP Recherche,** Parc Ester Technopole, 24 Rue Atlantis - Bat Boréal, 87069 Limoges, France

**Keywords:** Automated telesurveillance system, Falls, Nursing homes, Secondary prevention, Older adults

## Abstract

**Introduction:**

Early detection and management of falls is an important issue in nursing homes, and automated telesurveillance system (ATS) can help support these processes. The main aim of this study was to examine the impact of ATS on serious falls prevention in older adults (OA) living in Nursing Home (NH).

**Materials and methods:**

This was a prospective, multicenter, randomized clinical trial. It involved 213 OA, 104 in the intervention group (IG) and 109 in the control group (CG).

**Results:**

The OA had a mean age of 87.7 ± SD: 5.9 years, and were predominantly female (69.0 %). The annual incidence of serious falls was 0.37 ± SD: 0.76, respectively 0.24 ± SD: 0.53 and 0.49 ± SD: 0.92 in the IG and CG, *p* = 0.022. That for non-serious falls was 0.97 ± SD: 1.98, respectively 0.84 ± 2.04 and 1.09 ± SD: 1.91 in the IG and CG, *p* = 0.011. The incidence of total falls was 1.33 ± SD: 2.48, 1.08 ± SD: 2.35 in the IG and 1.58 ± SD: 2.58 in the CG, *p* = 0.004. The proportion of OA with at least one serious fall was 20.19 % in the IG versus 33.03 % in the CG, *p* = 0.034. The proportion of OA with at least one non-serious fall was 25.96 % in the IG versus 45.87 % in the CG, *p* = 0.002.

**Conclusions:**

The ATS EDAO detects falls that are not serious, enabling personalized secondary prevention programs to be initiated to prevent serious falls.

## Highlights

• This is a randomized controlled trial.
• This research is registered in http://www.clinicaltrials.gov/ on 03/07/2012 under the number NCT01551121.
• She studied the impact of Automated Telesurveillance System (ATS) in preventing serious falls in nursing homes.

## 1 Introduction

The risk of falling is particularly high in nursing homes. The incidence of falls in these establishments is 1.7; 95 % CI: (0.6; 3.6) per year [1,2]. This incidence is higher than that observed in older persons living at community 0.65; 95 % CI: (0.3; 1.6) [1,3]. 46 % of falls in nursing homes occur at night [2]., and many of these go undetected by healthcare professionals, causing serious deterioration in the already fragile health of older adults living in these establishments. About 4 % of falls in nursing homes result in fractures. Head injuries, soft tissue injuries, severe lacerations or other serious trauma occur in around 12 %, 95 % CI: (1 %; 36 %) of cases. Among older adults aged 85 and over, one in five fatal falls occurs in a nursing home [4]. In 10 % of cases, the time spent on the ground due to fall-related injuries exceeds one hour [5,6]. The consequences of prolonged ground stays include dehydration, malnutrition and reduced functional autonomy. In extreme cases, they can even lead to death [7]. A fall resulting in a prolonged stay on the ground of more than an hour increases the mortality rate by 50 % in the 6 months following the fall [7]. In addition, the late detection of falls increases the workload of healthcare professionals working in nursing homes. The emotional impact of falls by older adults on nursing staff is mentioned by Rush et al. (2009) [8]. These authors report that nurses often feel guilty when an older adults falls. This guilt leads them to doubt their ability to provide quality care. This observation was also made by Kim (2017) [9]. Apart from the human consequences, falls by older adults cost the community. A study conducted in France in 2021 highlights that the cost of a fall depends on its severity. It ranges from €9285.71; 95 % CI: (€6768.88; €12,073.14) to €2316.74; 95 % CI: (€1395.00; €3456.87) [10].

With the French population ageing and the number of older adults over 65 set to rise by 2.4 million by 2030, there is an urgent need to take action to prevent falls and reduce their severity [11]. Preventing falls in older adults has been a subject of research for over 40 years [12,13]. Prevention activities exist in several disciplines: physiotherapy, occupational therapy, nursing, geriatrics and gerontology. The importance of these disciplines for prevention is justified by the fact that 50 % of falls are avoidable [14]. There are many technological systems [15] used in nursing homes to prevent falls, detect them and alert carers in the event of a fall [15,16]. Most of these technologies are reactive, helping to reduce the time spent lying on the ground and enabling rapid assistance to be given to the person who has fallen [17]. They can also be used to detect non-serious falls and prevent serious falls by setting up personalized prevention programs.This is typically the case with automated Telesurveillance System, known as EDAO. These technologies have proven their effectiveness in hospitals [[18], [19], [20], [21]]. However, there are few studies on the use of these technologies in nursing homes.

The main aim of this study was to examine the impact of automated telesurveillance system on serious falls prevention in older adults living in nursing homes.

## 2 Methods

### 2.1 Design of research

This was a prospective, multicenter, comparative, randomized clinical trial in two unblinded parallel groups conducted between March 2012 and March 2017.

**The intervention group** is made up of older adults benefiting from automated Telesurveillance System. Automated telesurveillance system was active at night, from 8 pm. to 7 a.m., 7 days a week. In the event of a fall, a message is sent to the healthcare professionals' work computers and business telephones. The latter could then intervene quickly to assist the older adults. Only the static image was visible. The system is automatically deactivated when healthcare staff enter the room and activate the “healthcare professional presence”. This ensures that healthcare professionals or visitors authorized to enter rooms equipped with the system are not filmed. It was only when it was necessary to better understand the circumstances and mechanism of the fall that the multi-disciplinary commission for access to images is called upon to authorize or not access to the sequence of images recorded by the automated telesurveillance system. The investigating geriatrician could then review the images and implement corrections and/or a specific care plan depending on the cause identified.

**The control group** included older adults who did not have automated telesurveillance system in their rooms.

### 2.2 Ethics approval and consent to participate

The promoter and the investigators carried out this research in accordance with the French law n°2004–806 of August 9, 2004, as well as in accordance with the Good Clinical Practices (I.C.H. version 4 of May 1, 1996 and decision of November 24, 2006) and the declaration of Helsinki (Ethical principles applicable to medical research on human subjects, Tokyo 2004). This research is registered in http://www.clinicaltrials.gov/ on 03/07/2012 under the number NCT01551121. Older adults or their legal representatives have given their informed consent in writing. We therefore confirm that all research has been carried out in accordance with current guidelines and regulations.

### 2.3 Randomization

Randomization is carried out at the inclusion visit. It is stratified by center and will be performed electronically by connecting to the online platform of the “Unité Fonctionnelle de Recherche Clinique et Biostatistiques (UFRCB)” from the geriatrics clinical research unit at Limoges University Hospital. The list is compiled by the UFRCB in the form of blocks of variable size.

### 2.4 Eligibility criteria

The OA included in the study are aged 75 and over, and resided in one of the three nursing homes of Limoges, Peyrelevade and Gueret. In addition, they have given their clear and informed consent to participate in the study. A proxy (trusted support person, family member) could also give consent. Moreover, these older adults had to understand the study's objectives, respect the imperatives and complete the various assessments. Finally, they had to be able to get out of bed and have French health insurance.Older adults are excluded if they had a life-threatening illness in the short term (< 1 year) and if they lived in multiple rooms (if at least one older adults disagreed with the study).

### 2.5 Data collected

Data are collected three times: at inclusion, 6 and 12 months. The inclusion visit (M0 visit) was a comprehensive geriatric assessment. It included:

- Profile: age and gender
- Socio-environmental component: Previous occupation, educational level, family situation, legal protection.
- Assessment of functional autonomy with 4 scales: Activities of Daily Living (ADL) [22], Instrumental Activities of Daily Living (IADL) [23], Autonomie Gérontologie Groupes Iso-Ressources (AGGIR) » [24], and Functional Autonomy Measurement System (SMAF) [25].
- Cognitive assessment with: Mini Mental State Examination (MMSE) [26]
- Comorbidities and treatments.
- Sensory assessment listing visual, hearing and communication impairments.
- Assessment of thymic state with the 30-item Geriatric Depression Scale (GDS) [27]
- Nutritional assessment with the Mini Nutritional Assessment (MNA) [28] and the body mass index (BMI) [29].
- Assessment of physical capacity using the Fried criteria [30] and the Short Physical Performance Battery (SPPB) [31,32].
- Balance assessment with data on the history of falls in the last 12 months, the unipodal stance test [33] and the Timed up and Go test [34].
- Assessment of quality of life with the EQ5D3L (EuroQoL 5-Dimension 3-Level) scale [35].
- Other assessments: Alcohol and tobacco.

Follow-up visits took place at 6 and 12 months (± 30 days). They included the same elements as the inclusion visit and are carried out by the study's investigating physicians. During these visits, the investigator also collected the number and type of falls that had occurred since the last visit.

### 2.6 Measures

The primary outcome was the cumulative incidence of serious falls at 12 months in two groups. In addition, the incidence of non serious falls in the two groups is compared. The same was true for total falls.

The secondary objective was the proportion of older adults who fell seriously. In addition, a comparison is made between those who have had non-serious falls in the two groups. The same was true for older adults who had at least one fall (without distinguishing between serious and non-serious falls).

According to the World Health Organization (WHO), a fall is defined as an event in which a person inadvertently lands on the ground or any other surface at a lower level than before [36]. The definition of serious fall is based on the criteria of the French National Authority for Health (FNAH.) (i.e. presence of physical trauma, pressure sores, inhalation pneumonitis, dehydration, ground stay greater than 1 hour, rhabdomyolysis, hypothermia, hyperthermia, post-fall syndrome, focal neurological signs, increased frequency of falls) [37], excluding the criterion of "more than one hour on the ground", because in the control group it was difficult to know precisely how much time the older adult spent on the ground following the fall.

### 2.7 Sample size and statistical analysis

The number of older adults required for the study is calculated on the basis of the following assumption. At the time, there was no epidemiological study of the frequency of serious falls "établissement d'hébergement pour personnes âgées dépendant (EHPAD)" as defined by the FNAH. However, we chose the study by Nyberg et al. (1997) [38] because the nursing home population analyzed in this study was the closest to the EHPAD population. This is why the article uses the term “nursing home” instead of “EHPAD”. In this study, the incidence of serious falls was 38 %. The study showed similar adjusted results, with an odd ratio (OR) = 0.49; 95 % CI: (0.37; 0.65) using multi-domain fall prevention in nursing home [39]. With an annual incidence of serious falls of 38 % [38], and assuming a 50 % reduction in fall risk, an α risk of 0.05 and a β risk of 20 %, the calculated group size was 98 older adults. Considering the risk of secondary exclusion, 108 older adults are included per group. The number of older adults required for the study was therefore 216 (calculated using NQuery Advisor v7.0 software) [40].

Quantitative and qualitative variables are presented as means ± standard deviation (SD) and frequencies with proportions, respectively. Comparisons between quantitative variables are made using the Student's or Mann-Whitney U test. For qualitative variables, we have used the chi-square or Fisher test.

The level of significance in the study was set at *p* < 0.05. All statistical analyses are performed using SigmaStat 3.5 (SigmaStat, San Jose, CA, USA).

## 3 Results

### 3.1 Flowchart of older adults in the study

**Fig. 1:**
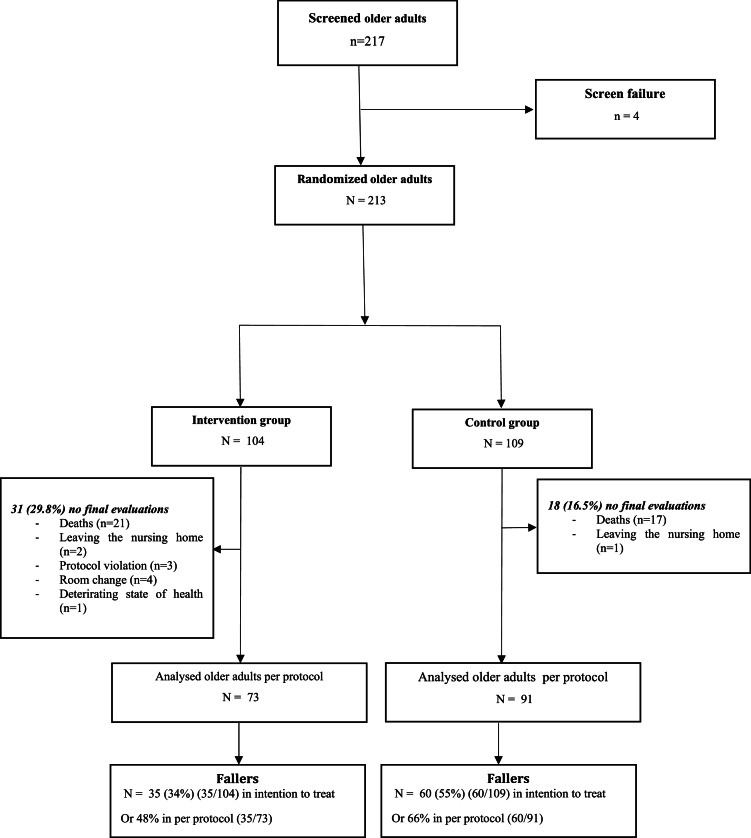
Flowchart of the TELEHPAD randomized controlled trial.

A total of 217 older adults were eligible for study. Of these, 213 are randomly assigned to either the intervention group (*n* = 104) or the control group (*n* = 109). Finally, the study included 164 older adults: intervention group (*n* = 73) and control group (*n* = 91). There were several reasons for exclusion. In the intervention group, there were five reasons for exclusion: death (*n* = 21), OA having changed rooms (*n* = 4), breach of protocol (*n* = 3), leaving the institution (*n* = 2) and deterioration in the older adults' state of health (*n* = 1). On the other hand, in the control group, there were two: deaths (*n* = 17) and older adults who left the nursing home (*n* = 1). During the 12 months of observation, 35 of the 73 older adults in the intervention group fell at least once. 60 of the 91 older adults in the control group also fell at least once during the same period (Fig 1).

### 3.2 Characteristics of the older adults in the study

Table 1 shows population characteristics. The average age of the older adults was 87.7 ± SD: 5.9 years, and they were predominantly female (69.0 %) and widowed (64.3 %). 18.3 % had worked for companies during their working lives. 75.6 % of the older adults in the study had no legal protection during their stay in a nursing home.

**Table 1:** Baseline characteristics of the TELEHPAD Study population, TELEHPAD randomized controlled trial.

Characteristics	Population *N* = 213	Intervention group *N* = 104	Control group *N* = 109	p-value
Age (years), mean ± SD	87.7 ± 5.9	87.8 ± 6.0	87.6 ± 5.9	0.848
Gender (female), n ( %)	147 (69.0)	70 (67.3)	77 (70.6)	0.124
Marital status (Widow (er)), n ( %)	137 (64.3)	67 (64.4)	70 (64.2)	0.118
Previous occupation (Compagny employee), n ( %)	39 (18.3)	18 (17.3)	21 (19.3)	0.986
Legal protection (None), n ( %)	161 (75.6)	83 (79.8)	78 (71.6)	0.051
Functional independance				
- ADL (score), mean ± SD - IADL (score), mean ± SD - SMAF (score), mean ± SD - GIR 2, n ( %)	2.7 ± 1.8 0.7 ± 1.0 49.3 ± 14.8 73 (34.3)	2.8 ± 1.9 0.8 ± 1.1 49.0 ± 15.6 29 (27.9)	2.7 ± 1.7 0.7 ± 1.0 50.1 ± 13.9 44 (40.4)	0.935 0.406 0.588 0.821
MMSE (score), mean ± SD	16.4 ± 6.9	16.7 ± 7.6	16.2 ± 6.1	0.499
Normal eye examination (no), n ( %)	163 (76.5)	76 (73.1)	87 (79.8)	0.932
Normal hearing examination (no), n ( %)	119 (55.9)	56 (53.8)	63 (57.8)	0.614
Normal thymic examination (yes), n ( %)	128 (60.1)	58 (55.8)	70 (64.2)	0.488
Normal nutritional examination				
- Malnutrition risk, n ( %) - MNA (score), mean ± SD - BMI (score), mean ± SD	128 (60.1) 21.5 ± 3.7 25.8 ± 4.7	61 (58.7) 21.4 ± 3.8 25.1 ± 4.0	67 (61.5) 21.6 ± 3.6 26.4 ± 5.2	0.270 0.470 0.070
Assessment of the balance				
- History of falls (no), n ( %) - Unipodal support < 5 s (yes), n ( %) - Time and go test > 20 s (yes), n ( %)	121 (56.8) 169 (79.3) 164 (77.0)	60 (57.7) 86 (82.7) 85 (81.7)	61 (56.0) 83 (76.1) 79 (72.5)	0.247 0.918 0.172
Assessment of the balance				
- EQ-VAS (score), mean ± SD - Utility (score), mean ± SD	53.3 ± 19.8 0.2 ± 0.5	53.0 ± 19.9 0.2 ± 0.5	53.7 ± 19.8 0.2 ± 0.5	0.687 0.980
- Fried criteria (score), mean ± SD	3.0 ± 1.0	3.0 ± 1.1	2.9 ± 0.9	0.425
Normal pulmonairy examination (yes), n ( %)	172 (80.7)	85 (81.7)	87 (79.8)	0.345
Normal neurological examination (yes), n ( %)	115 (54.0)	51 (49.0)	64 (58.7)	0.335
Normal locomotor examination, (yes), n ( %)	107 (50.2)	43 (41.3)	64 (58.7)	0.780
Normal abdominal system (yes), n ( %)	197 (92.5)	95 (91.3)	102 (93.6)	0.883
Normal skin condition, (yes), n ( %)	165(77.5)	82 (78.8)	83 (76.1)	0.905
Pathology (score), mean ± SD	8.1 ± 3.8	8.3 ± 4.1	7.9 ± 3.5	0.706
Treatment (score), mean ± SD	16.9 ± 7.1	16.6 ± 6.8	17.3 ± 7.3	0.473
Cardiovascular risk factors				
- HTA (yes), n ( %) - Diabetes (no), n ( %) - Dyslipidemia (no), n ( %)	136 (63.8) 174 (81.7) 163 (76.5)	65 (62.5) 91 (87.5) 80 (76.9)	71 (65.1) 83 (76.1) 83 (76.1)	0.670 0.725 0.346

In terms of functional autonomy, the average SMAF score was 49.3 ± SD: 14.8, reflecting low autonomy. The mean ADL and IADL scores were 2.7 ± SD: 1.8 and 0.7 ± SD: 1.0 respectively. In terms of dependency, older adults classified as GIR 2 are the most represented, accounting for 34.3 % of the total.

The mean MMSE score was 16.4 ± SD: 6.9. In terms of sensory status, examinations revealed that 76.5 % of older adults had eye problems and 55.9 % had hearing problems.

The examinations also revealed that thymic disorders, pulmonary problems, neurological problems and skin problems were less prevalent, with 60.1 %, 80.7 %, 54.4 % and 77.5 % respectively of older adults free of these problems. The same was true of locomotor problems and abdominal disorders, with 50.2 % and 92.5 % of older adults respectively free of these health problems.

In terms of nutritional status, the average MNA score was 21.5± SD: 3.7 and the average BMI 25.8 ± SD: 4.7. 60.1 % of the older adults were at risk of malnutrition.

The mean VAS-QE was 0.53 ± SD: 19.8 and the mean utility score following French weightings was 0.2 ± SD: 0.5.

In terms of physical capacity, the average frailty score according to Fried's criteria was 3.0 ± SD: 1.0.

Older adults had an average of 8.1 pathologies and 16.9 concomitant treatments. These included hypertension (63.8 % of patients), diabetes (18.3 %) and dyslipidemia (23.5 %).

### 3.3 Incidence, location and timing of falls

A total of 284 falls are recorded in two groups. In the intervention group, the total number of falls was 112, compared with 172 in the control group.

The annual incidence of serious falls was 0.37 ± SD: 0.76, respectively 0.24 ± SD: 0.53 and 0.49 ± SD: 0.92 in the intervention group and control group, *p* = 0.022.

The annual incidence of non-serious falls was higher than that of serious falls. It was 0.97 ± SD: 1.98. In the intervention group, the incidence of non-serious falls was 0.84 ± 2.04 and 1.09 ± SD: 1.91 in the control group, *p* = 0.011.

The incidence of total falls was 1.33 ± SD: 2.48, 1.08 ± SD: 2.35 in the intervention group and 1.58 ± SD: 2.58 in the control group, *p* = 0.004.

233 (81.04 %) falls occurred in the bedrooms of older adults, 134 (47.18 %) were nocturnal falls.Table 2

**Table 2:** Incidence, location and timing of falls at 12 months, TELEHPAD randomized controlled trial.

	**Population** ***N* = 284**	**Intervention group** ***n* = 112**	**Control group** ***n* = 172**	**p-value**
**INCIDENCE**				
Serious falls, mean ± SD	0.37 ± 0.76	0.24 ± 0.53	0.49 ± 0.92	0.022*
Non-serious falls, mean ± SD	0.97 ± 1.98	0.84 ± 2.04	1.09 ± 1.91	0.011*
Total falls, mean ± SD	1.33 ± 2.48	1.08 ± 2.35	1.58 ± 2.58	0.004*
**LOCATIONS**				
Bedrooms, n ( %)	233 (82.04)	92 (82.14)	141 (81.98)	0.972
Common areas, n ( %)	51 (17.96)	20 (17.86)	31 (18.02)	0.972
**TIMING**				
Morning, n ( %)	57 (20.07)	19 (16.96)	38 (22.09)	0.292
Afternoon, n ( %)	80 (28.17)	28 (25.00)	52 (30.23)	0.338
Evening, n ( %)	13 (4.58)	7 (6.25)	6 (3.49)	0.276
Night, n ( %)	134 (47.18)	58 (51.79)	76 (44.19)	0.210

*: Statistically significant results.

### 3.4 Comparison of proportion of fallers

The proportion of older adults with at least one serious fall was 20.19 % (104 older adults) in the intervention group versus 33.03 % (109 older adults) in the control group, *p* = 0.034.

**Fig. 2:**
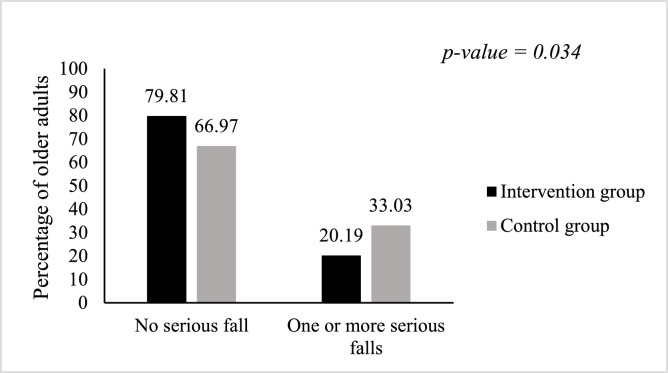
Proportion of older adults who fell seriously at least once in 12 months, TELEHPAD randomized controlled trial.

**Fig. 3:**
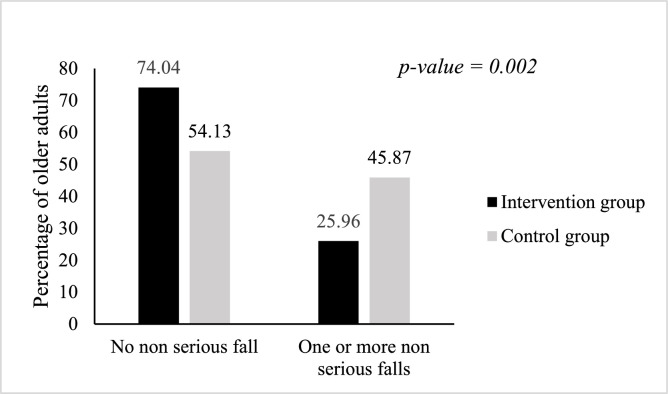
Proportion of older adults who had non-serious falls at least once in 12 months, TELEHPAD randomized controlled trial.

The proportion of older adults with at least one non-serious fall was 25.96 % (104 older adults) in the intervention group versus 45.87 % (109 older adults) in the control group, *p* = 0.002.Fig. 2, Fig. 3

## 4 Discussion

The aim of this study was to evaluate the impact of automated telesurveillance system in preventing serious falls in nursing homes. The sample comprised 213 older adults, 69.0 % of whom were women. Average age was 87.7 ± SD: 5.9 years.

The annual incidence of serious falls was 0.37 ± SD: 0.76. The annual incidence of non-serious falls was 0.97 ± SD: 1.98. Thus, the incidence of non-serious falls was greater than that of serious falls. The incidence of total falls was 1.33 ± SD: 2.48. The total incidence was similar to that observed in the study by Rubenstein et al. In that study, it ranged from 0.6 to 3.6 falls per older adults aged 75 and over living in nursing homes [3].

The importance of non-serious falls compared with serious falls reflects the effectiveness of personalized prevention programs after the first fall of an older adults in nursing homes has been detected. In fact, any fall detected immediately leads to rapid treatment to avoid worsening the consequences. In addition, an analysis of the fall is carried out by the healthcare staff, and a personalized secondary prevention program is initiated to prevent future serious falls.

In the control group, the incidence of non-serious falls was higher than in the intervention group, respectively 1.09 ± 1.91 and 0.84 ± 2.04, *p* = 0.011.

A study carried out in 2021 revealed that some healthcare professionals in nursing homes were skeptical about the ability of automated telesurveillance system to alert them in the event of a fall. Indeed, 76 % of them are not prepared to buy it for a relative in need. What's more, only 12 % of these professionals stressed that automated telesurveillance system was a support in their work. This observation is due to alerts received without a fall having taken place in the older adult's room [10].

This reluctance may lead professionals to focus their observations on the occurrence of falls in the control group. This will result in more falls being detected in this group than in the intervention group. This is a declarative bias, as it does not reflect the actual work of these healthcare professionals in the absence of automated telesurveillance system.

There are few studies on the use of automated telesurveillance system for prevention in nursing homes. Many studies exist on similar devices, but they are carried out in the context of their use with older adults in hospital or suffering from dementia. For example, Cournan et al. evaluated the effectiveness of automated telesurveillance system in preventing falls among older persons admitted to a 115-bed rehabilitation center, over a 21-month period. One year after the automated telesurveillance system was installed, the proportion of falls dropped significantly, from 6.34 to 5.10 falls per 1000 patient days [18].

82 % of all falls occurred in the bedrooms of older adults. The results of this study are in line with those of previous studies [41].

47.18 % of nocturnal falls are observed in both groups. Begoc also found a proportion of 41.6 % [42].

The proportion of fallers was higher in the control group. Indeed, for older adults who had at least one serious fall, the proportion was 33.03 % in the control group versus 20.19 % in the intervention group, *p* = 0.034. For older adults with at least one non-serious fall, the proportion was 45.87 % in the control group versus 25.96 % in the intervention group, *p* = 0.002.

One of the limitations already highlighted in the discussion was the change in the way healthcare professionals work in the presence of automated telesurveillance system. This change is likely to lead to a reporting bias in the number of falls in the control group.

This study also had several strengths.

It was a randomized study, which reduced the risk of selection bias or confounding, and ensured generalizability to the general population. It assessed the efficacy of automated telesurveillance system in preventing falls in nursing homes. This was an important initiative, given that many healthcare technologies are marketed without having been evaluated, especially in nursing homes. What is more, this study was one of the first to observe the impact of automated telesurveillance system in preventing falls in nursing homes. In addition, the size of the study was larger than in previous studies. Furthermore, the questionnaires are administered face-to-face to the older adults, providing comprehensive, high-quality information.

## 5 Conclusions

The incidence of falls was higher in the control group than in the intervention group. The differences observed were significant. Automated telesurveillance system appears to be an evidence-based strategy for the prevention of serious falls, insofar as it enables better detection of non-serious nocturnal falls and initiation of secondary prevention programs. The aim of the latter is to prevent the occurrence of future serious falls.

This technology is therefore a complementary tool that nursing home can use to support health professionals, who are understaffed at night. The “EDAO” automated telesurveillance system is currently marketed by TELEGRAFIK, which operates throughout France.

Further studies need to be carried out with healthcare staff to find out how they feel about using this technology. A similar study could be carried out with families and older adults themselves. In addition, some of the automated telesurveillance system imperfections need to be corrected to make it more effective. We also need to communicate with healthcare professionals so that they understand that this technology is a support, not a competitor.

## Funding information

This research has not received funding.

## Declaration of generative AI and AI-assisted technologies in the writing process

No artificial intelligence (AI) was used to write the manuscript.

## Conflict of interest declaration

The authors have declared no conflicts of interest with respect to the research, writing and publication of this article.

## CRediT authorship contribution statement

**Abdoul Razak SAWADOGO:** Writing – review & editing, Writing – original draft, Visualization, Validation, Supervision, Software, Methodology, Formal analysis, Conceptualization. **Jean-François NYS:** Writing – review & editing, Writing – original draft, Visualization, Validation, Conceptualization. **Caroline GAYOT:** Writing – review & editing, Writing – original draft, Visualization, Project administration, Formal analysis, Data curation, Conceptualization. **Gilles KEHOUA:** Writing – review & editing, Writing – original draft, Supervision, Software, Methodology, Formal analysis, Conceptualization. **Achille TCHALLA:** Writing – review & editing, Writing – original draft, Visualization, Validation, Supervision, Software, Resources, Project administration, Methodology, Investigation, Formal analysis, Data curation, Conceptualization.

## Declaration of competing interest

The authors have declared no conflicts of interest with respect to the research, writing and publication of this article.
